# Characterization of Actinomycetes Strains Isolated from the Intestinal Tract and Feces of the Larvae of the Longhorn Beetle *Cerambyx welensii*

**DOI:** 10.3390/microorganisms8122013

**Published:** 2020-12-16

**Authors:** Ramón I. Santamaría, Ana Martínez-Carrasco, Ricardo Sánchez de la Nieta, Luis M. Torres-Vila, Raúl Bonal, Jesús Martín, Rubén Tormo, Fernando Reyes, Olga Genilloud, Margarita Díaz

**Affiliations:** 1 Departamento de Microbiología y Genética, Instituto de Biología Funcional y Genómica (IBFG), Consejo Superior de Investigaciones Científicas (CSIC), Universidad de Salamanca, C/Zacarías González 2, 37007 Salamanca, Spain; anuskamc16@hotmail.com (A.M.-C.); ricardo.sancheznieta@usal.es (R.S.d.l.N.); 2 Servicio de Sanidad Vegetal, Consejería de Agricultura DRPyT, Junta de Extremadura, Avda. Luis Ramallo s/n, 06800 Badajoz, Spain; luismiguel.torres@juntaex.es; 3 Forest Research Group, INDEHESA, Escuela de Ingeniería Forestal, Universidad de Extremadura, Avda. Virgen del Puerto 2, 10600 Cáceres, Spain; raulbonal@unex.es; 4 Fundación MEDINA, Centro de Excelencia en Investigación de Medicamentos Innovadores en Andalucía, Avda. del Conocimiento 34, 18016 Granada, Spain; jesus.martin@medinaandalucia.es (J.M.); ruben.tormo@medinaandalucia.es (R.T.); fernando.reyes@medinaandalucia.es (F.R.); olga.genilloud@medinaandalucia.es (O.G.)

**Keywords:** actinomycetes, *Streptomyces*, *Amycolatopsis*, *Nocardiopsis*, *Cerambyx*, antibiotic production, hydrolytic enzymes

## Abstract

Actinomycetes constitute a large group of Gram-positive bacteria present in different habitats. One of these habitats involves the association of these bacteria with insects. In this work, we have studied twenty-four actinomycetes strains isolated from the intestinal tract and feces from larvae of the xylophagous coleopteran *Cerambyx welensii* and have shown that seventeen strains present hydrolytic activity of some of the following substrates: cellulose, hemicellulose, starch and proteins. Fourteen of the isolates produce antimicrobial molecules against the Gram-positive bacteria *Micrococcus luteus*. Analysis of seven strains led us to identify the production of a wide number of compounds including streptanoate, alpiniamide A, alteramides A and B, coproporphyrin III, deferoxamine, demethylenenocardamine, dihydropicromycin, nocardamine, picromycin, surugamides A, B, C, D and E, tirandamycins A and B, and valinomycin. A significant number of other compounds, whose molecular formulae are not included in the Dictionary of Natural Products (DNP), were also present in the extracts analyzed, which opens up the possibility of identifying new active antibiotics. Molecular identification of ten of the isolated bacteria determined that six of them belong to the genus *Streptomyces*, two of them are included in the genus *Amycolatopsis* and two in the genus *Nocardiopsis.*

## 1. Introduction

Cellulose, lignin, hemicellulose and chitin are the four most abundant polymers on Earth. While cellulose, lignin and hemicellulose are the main structural components of grassy and wood material, chitin is the polymer most abundant in the cell walls of fungi and the exoskeleton of arthropods (crustaceans and insects). Biological degradation is carried out mainly by fungi and bacteria, which produce the hydrolytic and oxidative enzymes used to recycle compounds, generating the different monomers or precursors that may be metabolized by the organisms themselves or by others. These decomposer microorganisms form part of complex ecosystems in which other higher organisms are sometimes implicated. Among these, insects are known to consume more lignocellulose than grazing mammals and include a wide number of cellulose-feeding species, of which termites have the most efficient degradation system [1].

Lignocellulose-feeding insects are grouped into several families including the family Cerambycidae (the so-called longhorn beetles) belonging to the order Coleoptera. Cerambycidae includes the genus *Cerambyx* and the two well-known species *C. welensii* and *C. cerdo*. Although these species are widely distributed, the ecological and legal considerations for these oak-living species are quite different. While *C. welensii* is considered a major inciting factor of oak decline in the Iberian peninsula, *C. cerdo* is included in the EU red list of endangered species and is currently protected under the Bern Convention (Council of Europe, 1979) and catalogued in Annexes II and IV of the EU Habitats Directive (Council of the European Communities, 1992) [2]. In some cases, *C. cerdo* has even been treated as an umbrella species in conservation programs [3]. In Spain, the main hosts of these two beetles are holm oak (*Quercus ilex* L.) and cork oak (*Quercus suber* L.), trees that form part of the so-called “Mediterranean dehesa” ecosystem, a unique type of open woodland with high environmental and socio-economic value [4]. The larvae of these beetles are strictly xylophagous while the adults feed mainly on sap and tree exudates. The larvae, on the other hand, have potent mandibles capable of chewing hard oak wood that permit them to create increasingly wider and longer galleries inside the trunk and main branches. These openings can allow pathogens to enter, leading to the structural and physiological damage that may cause the tree to eventually die [2,5].

Although some studies on the gut fluid of *C. cerdo* have identified enzymes capable of degrading filter paper, cotton and insoluble crystalline cellulose [1,6], so far there are no published reports on cellulolytic bacteria associated with the gut of *Cerambyx* species.

Actinobacteria have been isolated as cellulose degraders from the guts of termites and from *Sirex noctilio* (an invasive wood-feeding wasp) and other xylophagous insects [7,8]. In addition, in-depth studies on *Streptomyces* strains isolated from these habitats have shown that termite gut-associated actinobacteria produce secondary antimicrobial compounds that may be important for pathogen inhibition in the habitats of these insects [9]. Moreover, bombyxamycins A and B (1 and 2) are known to be produced from a *Streptomyces* strain in the gut of silkworms [10].

*Streptomyces,* and others actinobacteria, have also been isolated as suitable defensive partners associated with different insects [11], and it is known that *Streptomyces* and *Pseudonocardia* defend the fungal garden of leaf-cutting ants from pathogenic fungus [12]. Another example of this actinomycete–insect association can be found in different types of female beewolf wasps of the genera *Philanthus, Philanthinus* and *Trachypus* (Hymenoptera, Crabronidae) that cultivate *Streptomyces philanthi* in specialized antennal reservoirs and cover the wall of the cocoon to defend the larva from pathogens [13,14,15,16]. A study on *Streptomyces* sp. ICBG1318 isolated from *Melipona scutellaris* nurse bees, a stingless social insect, has led to the identification of two novel cyclic hexadepsipeptides, meliponamycin A (1) and meliponamycin B (2) [17].

Endophytic actinomycetes have also been isolated from a wide number of plants, where they may help to induce defense pathways. In fact, some produced compounds have been shown to exhibit antitumoral or anti-diabetic activities [18,19,20]. A tripartite mutualism between *Streptomyces*, strawberry plants and pollinating bees has been recently described, where pollinators transfer bacteria on flowers to new plants and the *Streptomyces* maintain the partnership by protecting the plants and insects against pathogens [21].

In this work, we have focused our study on the isolation of actinomycetes from the intestine and feces of *C. welensii* larvae. Isolation of several actinomycetes permitted us to study their capacity to produce cellulases, hemicellulases, amylases and proteases under laboratory conditions. The production of antibiotics and antifungals was also examined, and the findings showed that about 58% of the strains had the ability to produce different active molecules against Gram-positive bacteria. According to the LC/HRMS analyses of their extracts, some of the molecules produced, under laboratory conditions, were identified as streptanoate, alpiniamide A, alteramides A and B, coproporphyrin III, deferoxamine demethylenenocardamine, dihydropicromycin, nocardamine, picromycin, surugamides A, B, C, D and E, tirandamycins A and B, and valinomycin.

## 2. Materials and Methods

### 2.1. Larvae Collection and Dissection

Four mature *Cerambyx* sp. larvae were collected from a single *Quercus ilex* tree located in La Serrana, Mérida (SW Spain) at 39°01′22.8″ N 6°38′19.4″ W during the spring of 2017 and transported individually in sterile vials. These larvae were dissected as indicated by Hu et al. 2015 [22]. In brief, they were sterilized by immersion in 70% ethanol for 3 min and rinsed in sterilized water three times. Dissection was carried out using a stereomicroscope. The intestine was removed, suspended in sterile PBS buffer (137 mM NaCl, 2.7 mM KCl, 10 mM Na_2_HPO_4_, and 1.8 mM KH_2_PO_4_) and homogenized using sterile glass beads and a vortex. Since the larvae were kept in individual vials for 3 days before handling, all had produced feces that were also analyzed. The feces were suspended in PBS and vortexed until a homogeneous suspension was obtained. A piece of tissue from each larva was cut and frozen separately for subsequent identification (see below).

### 2.2. Bacterial Isolation and Media

Different dilutions of the mixed extracts obtained from the intestine and from the feces of the four *Cerambyx* larvae were individually spread on the surface of ISP2 [23] or R2YE plates [24] containing 25 µg/mL of nalidixic acid and 50 µg/mL of cycloheximide. The plates were incubated for two weeks at 28 °C and colonies with actinomycete phenotypes were selected and re-inoculated onto new plates of R2YE to obtain axenic cultures. All selected strains were maintained in the freezer at −80 °C in 20% glycerol until further use.

*Escherichia coli* DH5α, *Micrococcus luteus* (CECT 247) and *Saccharomyces cerevisiae* W303 2n were used for detecting the production of active compounds. LB medium was used to grow *E. coli* and YEPD for growing *M. luteus* and *S. cerevisiae* [25,26].

### 2.3. Enzymatic Activities Assayed

Cellulolytic activity was detected using solid NMMP plates [24] containing low viscosity 0.5% carboxymethylcellulose (CMC) (Sigma-Aldrich, St. Louis, MO, USA). Hydrolytic activity in the presence of paper was also assessed by using pieces of filter and 3MM paper in liquid NMMP, containing no other carbon source, grown at 28 °C for 3 days under agitation (200 rpm). Xylanolytic activity was detected using NMMP plates containing 0.5% oat spelt xylan (Sigma-Aldrich, St. Louis, MO, USA) amylase activity on NMMP plates containing 0.5% soluble starch (Scharlab S.L., Barcelona, Spain) and protease activity was assayed in the same solid medium containing 0.5% skimmed milk (Sveltesse, Nestlé S.A., Vevey, Switzerland). The different strains were inoculated by puncture and incubated at 28 °C for 4 days. Cellulase and xylanase activities were observed after flooding the Petri dish with 10 mL of 0.5% Congo red for 30 min and then washing the plates several times with 1M NaCl; amylase activity was detected by flooding the plates with lugol; protease activity was detected by precipitation of the unhydrolyzed skimmed milk using 10% trichloroacetic acid (TCA). All activities were observed as a clear halo around the colony growing at the site of inoculation.

### 2.4. Determination of Antimicrobial Production by the Actinomycetes Isolated

Antibiotic or antifungal production was studied by growing all strains on solid ISP2, R2YE and SFM plates at 28 °C for 8 days. Using a cork borer, a 0.7 cm disc of agar containing each microorganism was obtained and assayed on LB plates inoculated with a lawn of *E. coli* or on YEPD plates inoculated with a lawn of *M. luteus* or *S. cerevisiae.* Activity was observed after incubation at 30 °C for 1 or 2 days, depending on the organism used in the assay.

### 2.5. DNA Extraction and Identification of Cerambyx sp. Larvae

Larval DNA was extracted from tissue of the four larvae using the commercial kit E.Z.N.A.^®^ Tissue DNA (OMEGA BIO-TEK Inc., Norcross, GA, USA). Then, a 658-base pair (bp) fragment of the mitochondrial gene *COI* (Cytochrome Oxidase I) was amplified using the universal primer pair LCOI1490/HCOI2198 [27] (Supplementary Table S1). Sequencing was performed using Big-Dye (Perkin-Elmer Inc., Waltham, MA, USA) technology and an ABI3700 sequencer. Sequence chromatograms were assembled, visually inspected and edited using Sequencher 4.6 (Gene Codes Corp., Ann Arbor, MI, USA). The extreme ends of the sequences were trimmed to obtain a final length of 625 bp. DNA barcoding was done by aligning the sequences from all 4 larvae with those obtained for the same gene fragment from adult *C. cerdo* and *C. welensii*.

### 2.6. DNA Extraction and Identification of the Actinomycetes that Produce Bioactive Molecules

Total genomic DNA was obtained from each of the actinomycetes strains producing bioactive molecules from cell cultures grown in liquid TSB medium (Condalab, Madrid, Spain) at 28 °C for 24–36 h. PCR was carried out using the universal primers for 16S RNA, primer set 27F (5′-AGA GTT TGA TCM TGG CTC AG-3′) and 1525R (5′ AAG GAG GTG WTC CAR CC-3′), in a standard 50 μL reaction using Kapa high fidelity polymerase (Hoffmann-La Roche, Basel, Switzerland). The thermocycler conditions began with an initial denaturation step at 95 °C for 5 min followed by 30 cycles, each consisting of 20 sec at 98 °C, 15 sec at 61 °C for annealing, and 1.40 min at 72 °C for extension followed by a final extension at 72 °C for 5 min.

The sequences corresponding to 16S rDNA (around 1500 bp each one) were obtained using different universal primers (Supplementary Table S1) and deposited in GenBank (accession numbers are indicated in Figure 2 and in Table 3).

The 16S rDNA sequences obtained were compared with those included in the Bacterial 16S Ribosomal RNA Database (NCBI Ref Seq Targeted Loci Project) using the *megablast* algorithm. Phylogenetic analysis of the 16S rRNA sequences from the selected isolates, the closest relatives identified in the database and the model organisms *Streptomyces coelicolor*, *Streptomyces venezuelae* and *E. coli* (this last one was employed as an outgroup) was conducted using MEGA X v10.1.8 software [28], after previously carrying out an alignment using the *ClustalW* algorithm. The evolutionary history was inferred by using the maximum-likelihood method and the Tamura–Nei model [29]. An initial tree based on the heuristic search was obtained automatically by applying neighbor-joining and BIONJ algorithms to a matrix of pairwise distances estimated using the Tamura–Nei model. The topology with a superior log likelihood value was then selected. A discrete Gamma distribution was used to model evolutionary rate differences among sites (5 categories: parameter = 0.2727). The rate variation model allowed for some sites to be evolutionarily invariable (34.98% sites). The tree with the highest log likelihood (−6663.38) was drawn to scale, with branch lengths measured in the number of substitutions per site. The percentages of replicate trees in which the associated taxa clustered together in the bootstrap test (1000 replicates) are shown next to the nodes [30].

### 2.7. LC-HRMS Analyses and Dereplication

Petri dishes containing solid R2YE inoculated with a lawn of each of the different isolates producing antibiotic or antifungal activities, were incubated for 8 days at 28 °C and extracted with 30 mL of methanol under continuous shaking for 2 h. After centrifugation at 16,000× *g* for 10 min, an aliquot of 1mL of supernatant was dried in vacuo. The dry extracts were resuspended in 100 μL of methanol and LC-LRMS and LC-HRMS analyses were performed as previously described [31,32]. The dereplication of components was performed in an automated manner with both LRMS and HRMS described methodologies. The molecular formula of the peaks that were not dereplicated by either of the aforementioned procedures was interpreted using the HRMS spectra and was searched against the Dictionary of Natural Products (DNP v29.1 June 2020, CRC Press), the Natural Product Atlas (NP Atlas, Lininton Lab, Simon Fraser University, Canada https://www.npatlas.org/joomla/) and the Natural Products Activity & Species Source Database, (NPASS, v1.0, Faculty of Science, National University of Singapore http://bidd.group/NPASS/index.php), restricting the results to prokaryotes and confirming the correlation of the UV spectra when available, to determine if any peak could be putatively annotated or considered as a possible new natural product.

## 3. Results

### 3.1. Cerambyx Larvae Species Identification

Since the two congeneric species *C. cerdo* and *C. welensii* can co-occur in Iberian oak open woodlands, their identification at the larval stage is problematic. In addition, the larvae of both species cannot be differentiated according to morphological traits thus requiring the use of DNA barcoding. The 625-bp DNA fragment amplified from the mitochondrial gene *COI* of four larvae (Materials and Methods) was compared with the same gene fragment originating from adult *C. cerdo* and *C. welensii* obtained in a previous study [33]. Three of the four larvae shared the same haplotype and the other differed from the rest by just two bp (0.3%); all larvae were identified as *C. welensii*. Identification by barcoding was found to be reliable, as both haplotypes had already been recorded in *C. welensii* adults trapped in the same area [33]. Pairwise genetic divergence between larvae and adult reference sequences was therefore much lower than the 1% threshold set by the Barcoding of Life Data System (BOLD) for molecular species determination [34].

### 3.2. Actinomycetes Isolation and Enzymatic Assays

**Figure 1:**
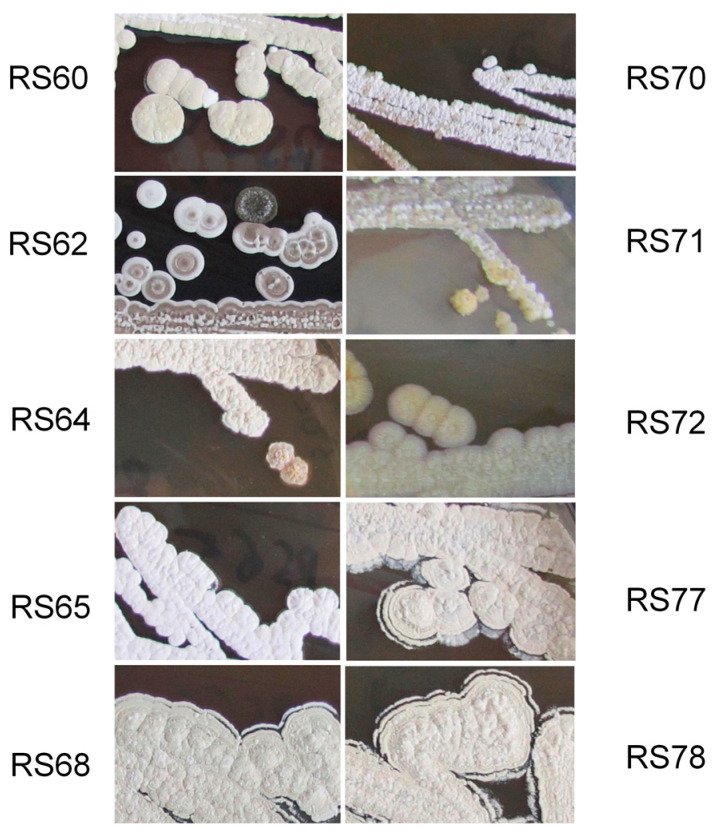
Morphology of the actinomycetes selected for molecular identification. The selected strains for molecular identification were isolated from intestine (RS60 and RS62) and from feces (RS64, RS65, RS68, RS70, RS71, RS72, RS77 and RS78). A portion of 3 cm of a normal petri dish is shown in each panel. The cultures were carried out on R2YE and incubated at 28 °C for 8 days.

In order to determine if these four *C. welensii* larvae hosted any actinomycete bacteria in their intestinal tract, different dilutions of mixtures of their intestines and feces were spread onto ISP2 and R2YE plates containing 25 μg/mL of nalidixic acid and 50 μg/mL of cycloheximide. After two weeks of incubation at 28 °C, 24 colonies with actinomycete morphological characteristics were selected. Three of the colonies were isolated from plates inoculated with the mixture of intestinal extracts and the remaining 21 colonies from plates inoculated using a feces mixture. All isolates were re-inoculated onto new R2YE plates to obtain axenic cultures and were conserved in 20% glycerol. The three strains isolated from the intestine were numbered RS60 to RS62 and those isolated from feces were numbered RS63 to RS83. A picture of 10 of the 24 isolated actinomycetes is shown in Figure 1.

The hydrolytic activity of all 24 isolates was studied on NMMP medium containing one of the following components: CMC, xylan, starch or skimmed milk (Material and Methods). It is worth noting that four of the isolates exhibited hydrolytic activity on all of the substrates tested. Isolate RS62, originating from the intestine, was found to be the most active of all of the strains (Table 1). An additional five isolates showed activity in the presence of substrates cellulose, xylan and starch, but not in the presence of skimmed milk. Nevertheless, none of these nine isolates exhibited activity with regard to filter or 3MM paper, which would have indicated cellulolytic activity in the presence of crystalline cellulose.

Additionally, four other isolates showed amylase and protease activities, one showed only amylase activity and three presented only protease activity. Therefore, 17 isolates exhibited some form of the hydrolytic activities assayed: 9 had cellulolytic activity, 9 had xylanolytic activity, 14 isolates had amylase activity and 11 strains had protease activity (Table 1).

Of the 24 actinomycetes isolated, seven did not present any of the hydrolytic activities assayed under laboratory conditions, of which one had been isolated from intestine (RS60) and the other six from feces (RS72, RS73, RS74, RS82, RS79, RS80) (Table 1).

### 3.3. Antibiotic and Antifungal Production and Compound Identification

The production of antibiotics and antifungals by the 24 isolates was also studied by growing these strains on solid ISP2, R2YE and SFM media and using *M. luteus, E. coli* and *S. cerevisiae* as the test organisms. The best production of active molecules was detected when the actinomycete strains were grown on R2YE. The assays employing *M. luteus* permitted us to observe that 14 of the 24 strains presented antibiotic activity against this Gram-positive bacterium. Meanwhile, only two strains (RS62 and RS64) produced a pseudo halo of growth inhibition in the presence of *E. coli.* No antifungal activity was detected against *S. cerevisiae* under the conditions assayed. Nine strains did not produce any antibiotic or antifungal molecules against the microorganisms assayed (Table 1).

The identification of the antimicrobials produced by seven of the strains was performed by LC-HRMS dereplication. A wide number of known molecules was detected in the MeOH extracts obtained from these species such as alpiniamide A, alteramides A and B, coproporphyrin III, deferoxamine, demethylenenocardamine, dihydropicromycin, nocardamine, picromycin, streptanoate, surugamides A, B, C, D and E, tirandamycins A and B and valinomycin (Table 2). Interestingly, several unidentified components were also obtained, which opens up the possibility of discovering new molecules with antibiotic activity.

### 3.4. Actinomycetes Strain Identification

The actinomycetes strains characterized in terms of antimicrobial production (Table 2) were identified based on their 16S rRNA sequences. The strains RS70 and RS71 were also included because the texture of their colonies was different to the typical *Streptomyces* colonies (Figure 1). The amplified 16S rDNA genes, of approximately 1500 bp, were sequenced and compared with the Bacterial 16S Ribosomal RNA Database (NCBI). The analysis (Table 3) showed that six of the isolates belonged to the genera *Streptomyces* (RS62, RS64, RS65, RS68, RS77 and RS78), two belonged to *Nocardiopsis* (RS60 and RS72) and two to *Amycolatopsis* (RS70 and RS71).

**Figure 2:**
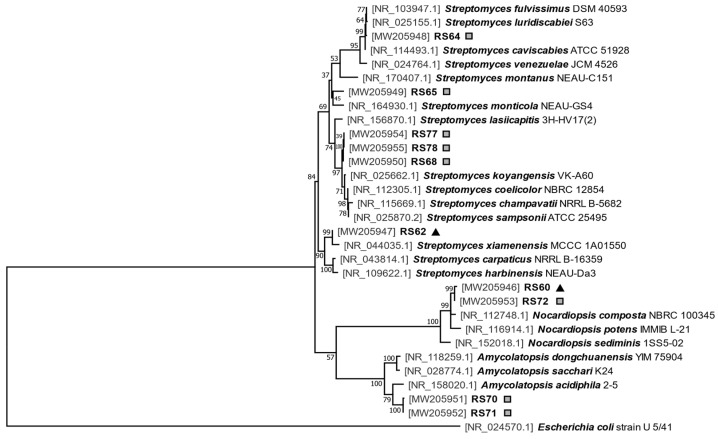
Phylogenetic analysis. Maximum likelihood phylogenetic tree generated using 16S rRNA sequences (the sequence of *E. coli* was used as the outgroup). Accession numbers are shown in brackets. Branch length is measured in number of substitutions per site (Scale Bar = 0.1). Bootstrap values based on 1000 replicates are shown next to the nodes. The actinomycetes isolates from the feces of *C. welensii* are marked with squares, while the isolates from the intestine are marked with triangles.

This analysis was support by a maximum-likelihood phylogenetic analysis. The tree with the highest log likelihood (−6663.38) is shown in Figure 2.

Several isolates clustered together in the 16S rRNA analysis and showed a similar phenotype (RS68, RS77 and RS78, RS60-RS72 and RS70 and RS71) (Figure 1) which indicates they are probably different strains of the same species. Furthermore, two similar isolates, RS60 and RS72, with high similarity (>97%) to *Nocardiopsis potens* IMMIB L-21 were isolated from intestine and feces, respectively, and produced different types of molecules, some of which were not identified in the DNP. These results support the need to use both sources (intestine and feces) in order to analyze the microbiota of the digestive tract of *C. welensi* larvae.

## 4. Discussion

The gastrointestinal tract of insects remains a largely unexplored habitat [11]. As far as we know, this is the first report describing microorganisms isolated from the intestinal tract and feces of the xylophagous larvae of *C. welensii.* We focused our research on the isolation and study of actinomycetes. After identification of the ten best antimicrobial producers, we have shown that six of them belong to the genera *Streptomyces,* two have been identified as *Amycolatopsis* and two as *Nocardiopsis*. Production of hydrolytic enzymes is normal in a large number of the actinomycetes originating from different sources [35]. In fact, actinobacteria associated with termites enhance the acquisition of nutrients from a diverse array of polysaccharides including cellulose [8,36]. In our research we observed that 17 of the 24 isolated bacteria exhibit one or several of the enzymatic activities studied, (cellulase, xylanase, amylase and protease), with four out of the 17 strains showing all activities. Moreover, strain RS62, isolated from intestine, was the one that had the strongest activity in the presence of most of the substrates assayed (Table 1). However, these results could be expected, owing to the habitat of the larvae included in this study and their ability to feed on lignocellulosic material.

In addition, our assessment of the antibiotic production of these strains indicated that 14 out of the 24 isolates produce some antimicrobial compounds that are mainly active against Gram-positive bacteria. Only two of the strains (RS62 and RS64) produce pseudo halos on a lawn of *E. coli.* None of the 24 strains analyzed had a clear antifungal activity against *S. cerevisiae*, even in the case of strain RS77 which is known to produce the antifungal compound alteramide A; this was perhaps due to a low level of production that did not allow its activity to be detected. Our results suggest that the actinomycete strains present in the guts of *Cerambyx* are protecting them from infection by Gram-positive bacteria, inhabitants of the same environment that could presumably be more abundant. It is also interesting to note that only four of the isolated strains, (RS73, RS74, RS79 and RS80) did not show hydrolytic activity or produce any active antibacterial or antifungal compounds under the conditions assayed.

Among the antibiotics identified, alpiniamide A is a linear polyketide known to be produced by endophytic *Streptomyces* bacteria. This antibiotic has an unexpected nonlinear synthesis that involves a hybrid polyketide synthase-nonribosomal peptide synthetase. It is assembled in two halves and is then ligated into the mature molecule [37,38]. Another compound produced by one of the isolated strains is the polyketide alteramide A. This molecule was identified previously from *Streptomyces puniceus* strain L75 (isolated from the rhizosphere of *Acanthus ilicifolius*) and can inhibit the fungal phytopathogens involved in tomato “Early blight disease”. Alteramide A production has also been detected by a *Pseudoalteromonas* sp. isolated from a healthy gorgonian octocoral that had antifungal activity against *Penicillium citrinum,* one of the fungi implicated in the massive destruction of some soft corals [39,40]. Deferoxamine is an iron chelator commonly produce by *Streptomyces* species that may have relevant applications in the growing field of tissue regeneration [41]. Demethylenenocardamine is a desferrioxamine-related compound that has been previously described in a *Streptomyces* sp. isolated from a marine sponge and from *S. clavuligerus* ATCC 27064 [42,43]. Nocardamine or desferrioxamine E was produced by four strains (RS62, RS64 RS68 and RS77) and was the most abundant antibiotic. This compound is a cyclic siderophore that has antitumor activity, previously described from a marine isolate, *Citricoccus* sp. KMM 3890, and from *Streptomyces* sp. Nocardamine-like compounds can be useful in the treatment of acute iron intoxication and in ecological remediation [44,45]. Additionally, germicidins are natural products that inhibit spore germination [46] and germicidin G has previously been described as being produced by *Streptomyces* endophytes and marine *Nocardiopsis* strains [47,48]. The production of the macrolides pikromycin and dehydropikromycin by *S. venezuelae* ATCC-15439 has also been described and has the potential to combat multi-drug-resistant respiratory pathogens [49,50]. Streptanoate is an anticancer butanoate isolated previously from *Streptomyces* sp. DC3 [51]. Surugamides A and E are compounds that have been detected in conjunction with two of the *Streptomyces* strains isolated in this work (RS68 and RS77), they are cyclic octapeptides that are inhibitors of cathepsin B. Surugamide A is known to be produced by *S. albidoflavus* J1074, isolated from soil, and from *Streptomyces* sp. SM17 isolated from a marine sponge [52,53]. Tirandamycine A and B were previously identified from marine-derived actinomycetes and tirandamycine A has been described as having antiamoebic properties against *Entamoeba histolytica.* Tirandamycine B was also isolated from *Streptomyces* sp. 17944 and can kill the nematode *Brugia malayi* in the adult stage, which causes human filariasis [54,55]. The ionophore valinomycin, on the other hand, is a respiratory chain uncoupler that activates mitophagy via the PINK1/Parkin signaling pathway and plays an important role in clearing dysfunctional mitochondria through mitophagy in people with Parkinson’s disease [56]. Interestingly, some of the compounds produced do not present any clear correspondence in the DNP. Thus, some of the molecules produced by the *Nocardiopsis* strain RS60 have formula that are coincident with known molecules like benanomicin C (C_29_H_25_NO_12_) and buanmycin (C_30_H_27_NO_12_); however, the UV spectra described for these molecules do not conform with what was observed. In this study, we have also detected a large number of molecules (C_13_H_18_O_5_; C_17_H_25_NO_4_; C_34_H_57_NO_13_; C_30_H_25_NO_11_; C_29_H_23_NO_11_) produced mainly by strain RS60, a *Nocardiopsis*, that do not correspond with molecules in the DNP, the NP Atlas nor the NPASS. These results suggest there are several novel bioactive molecules being produced that will require further characterization.

## 5. Conclusions

This work highlights the ecological importance of actinomycetes in the biology of *Cerambyx* larvae and, at the same time, has embarked upon the search for finding new bioactive molecules. The characterization of new molecules, originating from unexplored sources, such as the digestive tract of *C. welensii* larvae, may potentially be used in the fight against microbial antibiotic resistance.

## Figures and Tables

**Table 1:** Hydrolytic and antibiotic activities of the actinomycetes isolated from *Cerambyx welensii* larvae. (-): No activity; df: diffuse halo with non-sharp edges.

Collection Number	Origin	Halo of Hydrolytic Activities (Diameter: cm)				Antibiotic Activity (Diameter: cm)
		Cellulase	Xylanase	Amylase	Protease	*Micrococcus luteus*
RS60	Intestine	-	-	-	-	1.8 df
RS61	Intestine	-	-	3.0	2.0	-
RS62	Intestine	1.6	1.2	2.1	2.3	1.0
RS63	Feces	0.4	0.4	1.0	-	-
RS64	Feces	0.4	0.4	1.0	-	2.6
RS65	Feces	0.4	0.4	1.3	-	1.0
RS66	Feces	0.4	0.4	1.7	-	1.0
RS67	Feces	-	-	2.8	1.8	1
RS68	Feces	-	-	2.6	1.8	1.4
RS69	Feces	1.0	1.6	1.2	1.1	-
RS70	Feces	-	-	-	1.6	1.5 df
RS71	Feces	-	-	-	0.9	1.5 df
RS72	Feces	-	-	-	-	1.5 df
RS73	Feces	-	-	-	-	-
RS74	Feces	-	-	-	-	-
RS75	Feces	0.2	0.3	1.7	-	1.0 df
RS76	Feces	-	-	-	1.1	1.8 df
RS77	Feces	-	-	1.6	1.1	1.3
RS78	Feces	-	-	-	-	1.1
RS79	Feces	-	-	-	-	-
RS80	Feces	-	-	-	-	-
RS81	Feces	-	-	0.7	-	-
RS82	Feces	0.6	0.9	1.1	0.6	-
RS83	Feces	0.4	0.9	0.6	0.6	-

**Table 2:** Molecules produced by seven isolated actinobacteria.

Strain	Compounds Putatively Produced
RS60	Alpiniamide A, streptanoate, picromycin, valinomycin
RS62	Nocardamine
RS64	Demethylenenocardamine, nocardamine, N1-De-Ac, N1-phenylacetyl deferoxamine, N1-De-Ac, N1-phenylacetyl, N6-Ac-deferoxamine
RS65	Coproporphyrin III
RS68	Nocardamine, surugamide A and E, tirandamycin A and B, germicidin G
RS72	Picromycin, dihydropicromycin
RS77/RS78	Alteramide A and B, deferoxamine, nocardamine, N1-De-Ac, N1-phenylacetyl deferoxamine, surugamides A, B, C, D and E, tirandamycin A and B, germicidin G

**Table 3:** Analysis of 16S rRNA sequences. Results from the comparison of the 16S rRNA sequences of the selected isolates to the Bacterial 16S Ribosomal RNA Database (NCBI). Only the closest relative obtained in each case is shown.

Isolates			Closest Relative in the Bacterial 16S rRNA Database			
Acc. No.	ID	Source	Strain	Acc. No.	Identity	Query Cover
MW205946	RS60	Intestine	*Nocardiopsis potens* IMMIB L-21	NR_116914.1	97.94%	98%
MW205947	RS62	Intestine	*Streptomyces xiamenensis* MCCC 1A01550	NR_044035.1	99.13%	97%
MW205948	RS64	Feces	*Streptomyces luridiscabiei* S63	NR_025155.1	99.80%	100%
MW205949	RS65	Feces	*Streptomyces monticola* NEAU-GS4	NR_164930.1	97.70%	100%
MW205950	RS68	Feces	*Streptomyces sampsonii* ATCC 25495	NR_025870.2	99.28%	100%
MW205951	RS70	Feces	*Amycolatopsis acidiphila* 2-5	NR_158020.1	97.83%	97%
MW205952	RS71	Feces	*Amycolatopsis acidiphila* 2-5	NR_158020.1	97.89%	97%
MW205953	RS72	Feces	*Nocardiopsis potens* IMMIB L-21	NR_116914.1	98.40%	98%
MW205954	RS77	Feces	*Streptomyces sampsonii* ATCC 25495	NR_025870.2	99.21%	100%
MW205955	RS78	Feces	*Streptomyces sampsonii* ATCC 25495	NR_025870.2	99.21%	100%

## Supplementary Materials

The following are available online at https://www.mdpi.com/2076-2607/8/12/2013/s1, Table S1: Oligonucleotides used in this work.
