# Supplementary material for: Characterization of Actinomycetes Strains Isolated from the Intestinal Tract and Feces of the Larvae of the Longhorn Beetle Cerambyx welensii
Source: Microorganisms. 2020 Dec 16;8(12):2013. doi: 10.3390/microorganisms8122013 (PMC7766275; doi:10.3390/microorganisms8122013)
Supplement: Supplementary file 1 [file microorganisms-08-02013-s001.pdf]

Supplementary Table S1. Oligonucleotides used in this work.

Primer	Sequence (5′ - 3′)	Application
LCO1490	GGTCAACAAATCATAAAGATATTGG	Amplification of mitochondrial gene *COI*, Cytochrome Oxidase I (*Cerambyx*).
HCO2198	TAAACTTCAGGGTGACCAAAAAATCA	Amplification of mitochondrial gene *COI*, Cytochrome Oxidase I (*Cerambyx*).
27F	AGAGTTTGATCMTGGCTCAG	Amplification of 16S rDNA gene (bacteria).
1525R	AAGGAGGTGWTCCARCC	Amplification of 16S rDNA gene (bacteria).
SF1	AGAGTTTGATCMTGGCTCAG	Sequencing of 16S rDNA gene (bacteria).
SF3	GTGCCAGCMGCCGCGG	Sequencing of 16S rDNA gene (bacteria).
SF4	ATTAGATACCCTGGTAG	Sequencing of 16S rDNA gene (bacteria).
SF5	GCAACGAGCGCAACCC	Sequencing of 16S rDNA gene (bacteria).
SR2	GWATTACCGCGGCKGCTG	Sequencing of 16S rDNA gene (bacteria).
SR3	CCGTCAATTCMTTTRAGTTT	Sequencing of 16S rDNA gene (bacteria).
SR4	GGGTTGCGCTCGTTG	Sequencing of 16S rDNA gene (bacteria).
SR5	AAGGAGGTGWTCCARCC	Sequencing of 16S rDNA gene (bacteria).
